# Hybrid Orientation Based Human Limbs Motion Tracking Method

**DOI:** 10.3390/s17122857

**Published:** 2017-12-09

**Authors:** Grzegorz Glonek, Adam Wojciechowski

**Affiliations:** Institute of Information Technology, Faculty of Technical Physics, Information Technology and Applied Mathematics, Lodz University of Technology, 215 Wolczanska street, 90-924 Lodz, Poland; adam.wojciechowski@p.lodz.pl

**Keywords:** data fusion, depth sensor, Microsoft Kinect, IMU, motion tracking

## Abstract

One of the key technologies that lays behind the human–machine interaction and human motion diagnosis is the limbs motion tracking. To make the limbs tracking efficient, it must be able to estimate a precise and unambiguous position of each tracked human joint and resulting body part pose. In recent years, body pose estimation became very popular and broadly available for home users because of easy access to cheap tracking devices. Their robustness can be improved by different tracking modes data fusion. The paper defines the novel approach—orientation based data fusion—instead of dominating in literature position based approach, for two classes of tracking devices: depth sensors (i.e., Microsoft Kinect) and inertial measurement units (IMU). The detailed analysis of their working characteristics allowed to elaborate a new method that let fuse more precisely limbs orientation data from both devices and compensates their imprecisions. The paper presents the series of performed experiments that verified the method’s accuracy. This novel approach allowed to outperform the precision of position-based joints tracking, the methods dominating in the literature, of up to 18%.

## 1. Introduction

Human limbs motion tracking can be defined as a process of unambiguous limbs joints spatial positioning, devoid of significant delays. Nowadays, it might be exploited within several areas, such as entertainment issues, virtual environments interaction or medical rehabilitation treatment [1]. However, it is the system precision that determines its possible application.

Since 1973, when Gunnar Johanson invented his motion capture system [2], such techniques were available mainly for professionals. However, since 2010, when the Microsoft Company (Redmond, WA, USA) released the Kinect v.1 controller, motion capture became available to almost everyone. The Microsoft Kinect controller is an exemplary implementation of the optical, markerless depth sensor with the most remarkable human motion capture functionality. Though it was designed for, and mainly used in the field of home physical activity games, Kinect became a popular subject for many researchers, whose goal was to find other, more advanced scenarios, where this controller might be applied e.g., Lange et al. [3] and Chang et al. [4] designed Kinect based systems to support physical rehabilitation for people with motor disabilities.

Microsoft Kinect represents a broad group i.e. PrimeSense Carmine (PrimeSense, TelAviv, Israel), ASUS Xtion (AsusTek Computer Inc., Taipei, Taiwan) of mono-view markerless depth sensors that exploit structural light patterns to reconstruct the depth of the scene and track perceived objects, with special attention put to human limbs and body parts tracking (i.e., face tracking). This group reveals similar constraints and limits that were usually compensated by sophisticated algorithms of other modes controllers (i.e., inertial, magnetic, acceleration, etc.) [5].

An easy access to the motion capture has been additionally supplemented and magnified by the fact that inertial devices such as gyroscopes and accelerometers (IMU – inertial measurement units) have become an integral and mandatory part of current electronic devices. These units are built in almost every smartphone device and they are used in various applications like pedometers [6,7], screen rotators [8] or digital camera orientation detectors.

Inertial measurement units (IMU) are also used as measuring devices in non-optical, inertial human limbs motion tracking systems i.e., XSense [9,10]. Despite IMU sensors being used in products available for home users, and are being improved continuously, their measurements precision and work limitations can be recognized as blockers for motion capture scenarios that require high accuracy of joint positioning.

The broad and easy access to mentioned types of devices became a trigger for many researchers around the world to work on these controllers’ data fusion methods that would increase the accuracy of human skeleton joint positioning. Bo et al. [11] focused on the knee joint angle estimation. They proposed accelerometer and gyroscope data fusion, with linear Kalman filter, to calculate the angle, and then aligned it to the Kinect’s estimation. In this approach, Kinect’s measurements were treated as reference data so that means authors treated them as always reliable. This also means that the method can only work while Kinect measurements are available. In the case of occlusions or any other disorders, the proposed method will give incorrect results. Destelle et al. [12] have proposed the method based mainly on IMU devices. In this method, human skeleton bones’ orientations and overall pose of the body model have been estimated basing on inertial data only, while sizes of skeleton bones were set basing on Kinect estimation. In addition, the skeleton placement was defined by the *Spine* joint position of the Kinect’s originated skeleton model. The data from the accelerometer, gyroscope and magnetometer were fused with Madgwick’s filter [13]. In Destelle’s method, the role of the Kinect was reduced to only two functions: measuring the initial length of bones and tracking the location of the user (its selected joint position) on the scene. The pose of the user was estimated only by IMU. Unfortunately, authors didn’t publish results that would allow for defining the accuracy of that method. Kalkbrenner et al. [14] proposed combining together Madgwick’s filter with the linear Kalman filter. In their approach, they fused accelerometer and gyroscope data with Madgwick’s filter to estimate the selected bone rotation. Basing on this information and on the bone length, estimated with Kinect, joint positions were calculated. In Kalkbrenner’s approach, bone lengths were taken from Kinect’s data in every measurement frame. This means that this value was dynamic and may differ even a few centimeters between two consecutive frames. In the last step, joint positions, estimated by Kinect, were fused by means of the linear Kalman filter with joint positions, estimated simultaneously with IMU sensors. Kalkbrenner’s method was the method with the highest declared precision of the joint position estimation. Published results declare the position estimation error of about 2.2 cm; however, the method was tested on sensors’ privileged body gestures. In the method proposed by Feng and Murray-Smith [15], the accelerometer data has been fused with Kinect measurements with the linear Kalman filter, modified by authors to work with frequency unaligned signals. Publicly available and described results of the Murray-Smith method show that the proposed approach allows for stabilizing joint position estimation, at the beginning or at the end of the movement, much faster than the classical Kalman filter implementation. On the other hand, published diagrams show results in a short time period (no longer than 5 s), so it is difficult to retrieve the joint positioning accuracy for this method. Tannous et al. [16] proposed the extended Kalman filter to fuse bone rotations estimated both by Kinect and IMU devices. Their experiments focused on the knees flexion in fully visible position without any occlusion. The elaborated error for knee angle estimation was $6.79{}^{\circ }$ for the left knee and $8.98{}^{\circ }$ for the right knee. The last considered method that addresses a similar problem of upper limb motion tracking with IMU and Kinect devices is the one described in the Tian et al. [17] article. Authors of this method proposed the usage of Unscented Kalman Filter (UKF) for both accelerometer and gyroscope data fusion as well as IMU and Kinect fusion. The uniqueness of the proposed method was taking into consideration geometrical constraints of the human pose. If, according to mentioned constraints, the estimated pose was impossible to achieve, the estimation was aligned to the closest, valid value. Thanks to that approach, authors achieved deviation of rotation angle estimation lower than $20{}^{\circ }$.

The analysis of the available literature shows that the majority of known data fusion methods is based on different variants of Kalman filter (linear, extended or unscented), to fuse accelerometer with gyroscope data as well as IMU data with these provided by the Kinect device. Only Kalkbrenner et al. decided to fuse accelerometer and gyroscope data with Madgwick’s filter, which is designed especially for this kind of data and, regarding the Madgwick’s report [13], it has better accuracy and performance than the linear Kalman filter. It is also noticeable that authors of these methods are selectively taking into consideration characteristics and limitations of measurement devices. As an example, Bo et al. [11] and Destelle et al. [12] focused mainly on IMU devices and their intrinsic data fusion, treating Kinect device data as a reliable reference for their methods, despite the known inaccuracy of this device. Kalkbrenner et al. [14] as well as Feng and Murray-Smith [15] tried to compensate some Kinect’s measurement problems using proper parameters in the Kalman filter. However, they focused only on joint position estimation fluctuations, e.g., Kalkbrenner recalculated fusion parameters only when the difference in the selected joint position estimation value, between two consecutive measurement frames, was greater than 15 cm. None of the authors included in their methods any compensation for IMU and Kinect’s measurement inaccuracy due to the environment conditions (the device operational temperature) or the context of the performed motion (the body rotation angle to the camera, the distance from the measurement device). The fact that these kinds of factors influence noticeably the quality and the accuracy of data measured by both devices may have an impact on the further data processing and on the final joint’s position estimation. Authors of the discussed methods fuse the signals of both measuring devices basing on joint positions and they don’t take bone orientations into consideration during that process. This fact has an impact on the final results, as joint positions used during the fusion include an additional, significant error caused by the non-fixed bones model length. The data fusion based on bone oation is free of this kind of error and it uses data that are measurable directly by both considered devices.

This paper presents the novel approach to depth sensor (Microsoft Kinect) and IMU data fusion that systemically makes up for limitations of both measurement devices and compensates their imperfections. The novel orientation based, human limbs’ joint positions tracking method outperforms state-of-the-art hybrid systems’ accuracy by 11–18%.

## 2. Materials and Methods

### 2.1. Microsoft Kinect Controller Characteristics

The Microsoft Kinect controller might be described as an RGB-D (Red, Green, Blue and Depth) camera. The first version of this device, originally designed for the Xbox 360 video game console, was built from two CMOS (Complementary metal–oxide–semiconductor) cameras and an integrated infrared (IR) projector. One of these CMOS cameras was responsible for an RGB signal and the second one was calibrated to record IR beam’s distribution. Considering the limbs’ motion tracking and the body gesture recognition, the most crucial part of Microsoft Kinect controller is the chip made by the PrimeSense company (PrimeSense, TelAviv, Israel). All recognition algorithms that stand behind Kinect’s motion tracking were implemented as a firmware for this processing unit. The Microsoft Company has not revealed any detailed description of tracking algorithms and key characteristics, so their retrieval became a subject for scientists and hobbyists [18,19,20]. The good understanding of the Kinect controller constraints seems to be indispensable to create an accurate method that would fuse its data with data collected from any other source.

**Figure 1:**
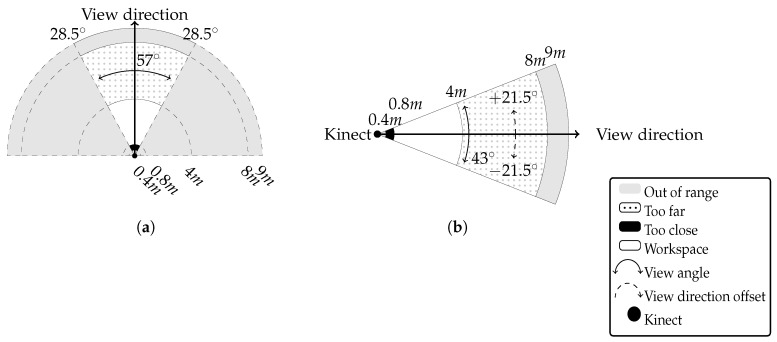
Microsoft Kinect v.1 operating range diagram in horizontal (**a**) and vertical (**b**) directions.

In the official device specification, it is claimed that the operating range of Kinect varies between 0.8 m and 4 m with the field of view $57{}^{\circ }$ horizontally (Figure 1a) and $43{}^{\circ }$ vertically (Figure 1b). At the same time, the specification does not reveal the possible heterogenity of the measurement’s accuracy. However, the experiments conducted by the authors showed that there are distance measurement nonlinearities within the Kinect’s working area. Moreover, researchers [21] and hobbyists [22] reported that the operating range is different among device series.

**Figure 2:**
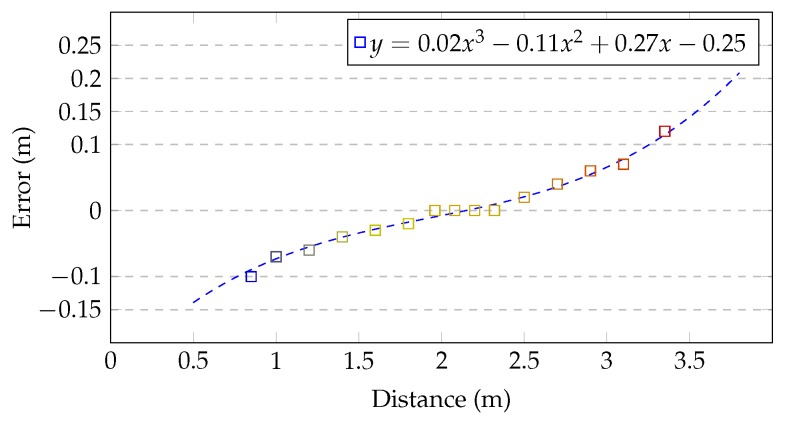
Microsoft Kinect depth measurement accuracy as a function of Kinect–joints distance.

A comparison of fluently changing distances between Kinect and limbs’ joints, collected simultaneously from Microsoft Kinect controller and Vicon (Vicon Motion Systems, Inc., Los Angeles, CA, USA) (Vicon—high accuracy, reliable, optical marker-based motion capture system) motion capture system, showed that Kinect has tendencies to underestimate the distance in the close range and to overestimate it in the far range. Collected values (squares on Figure 2) allowed to elaborate a distance estimation error function in a form of 3rd order polynomial, as it is presented in Equation (1), calculated based on Equation (2), where *X* is the matrix of sample points, *Y* is the matrix of values in these sample points and *A* is a coefficients matrix used in Equation (1):

(1) $$f\left(z\right)=0.02z^{3}-0.11z^{2}+0.27z-0.25,$$

(2) $$\begin{array}{cc} X^{T}XA & =X^{T}Y=>{\left[\begin{array}{cccc} x_{1}^{0} & x_{1}^{1} & x_{1}^{2} & x_{1}^{3}\\ x_{2}^{0} & x_{2}^{1} & x_{2}^{2} & x_{2}^{3}\\ x_{3}^{0} & x_{3}^{1} & x_{3}^{2} & x_{3}^{3}\\ \cdots \\ x_{n}^{0} & x_{n}^{1} & x_{n}^{2} & x_{n}^{3} \end{array}\right]}^{T}\left[\begin{array}{cccc} x_{1}^{0} & x_{1}^{1} & x_{1}^{2} & x_{1}^{3}\\ x_{2}^{0} & x_{2}^{1} & x_{2}^{2} & x_{2}^{3}\\ x_{3}^{0} & x_{3}^{1} & x_{3}^{2} & x_{3}^{3}\\ \cdots \\ x_{n}^{0} & x_{n}^{1} & x_{n}^{2} & x_{n}^{3} \end{array}\right]\left[\begin{array}{c} a_{0}\\ a_{1}\\ a_{2}\\ a_{3} \end{array}\right]={\left[\begin{array}{cccc} x_{1}^{0} & x_{1}^{1} & x_{1}^{2} & x_{1}^{3}\\ x_{2}^{0} & x_{2}^{1} & x_{2}^{2} & x_{2}^{3}\\ x_{3}^{0} & x_{3}^{1} & x_{3}^{2} & x_{3}^{3}\\ \cdots \\ x_{n}^{0} & x_{n}^{1} & x_{n}^{2} & x_{n}^{3} \end{array}\right]}^{T}\left[\begin{array}{c} y_{0}\\ y_{1}\\ y_{2}\\ \cdots \\ y_{n} \end{array}\right]. \end{array}$$

Figure 2 presents the plot of function defined with Equation (1). The analysis of this chart shows that Kinect’s distance estimation is optimal when the user stands about 2 m from the device (2–2.3 m). Outside this distance range, when Kinect works with a noticeable error, the systematic sensor depth correction should be taken into consideration.

Such inaccuracy and its tendencies are probably caused by the algorithm implemented in the Kinect controller. Though Microsoft did not reveal any precise technical description of their method for measuring distance between the camera and objects at the scene, original patent forms [23,24,25] owned by PrimeSense, combined with independent research results [26], provide some general overview of how this recognition algorithm works. It is known that the analyzed scene, in front of the Kinect controller, is enlightened with IR light dots structured pattern, which is skew symmetric, so the Kinect can work upright or upside down. Then, Kinect analyses the IR pattern’s dots distortion, and, basing on that, it estimates the subject distance. For the depth map reconstruction, Kinect uses two techniques in parallel: dots blurriness analysis [27] and stereo-vision based on a single IR camera and a projector [28]. The achieved depth map is a foundation of a human skeleton estimation algorithm. Depth-retrieved human body parts estimations are subsequently classified into body poses by means of the machine learning approach: random decision forest algorithms as well as object detection algorithms, such as the one proposed by Viola-Jones [29,30].

As the only source of data for that estimation process is an IR camera (RGB camera is not used at all), the observed scene must be free of any external source of the IR light. This requirement restricts the usage of Kinect in outdoor scenarios. A need for the scene isolation from any IR sources other than the Kinect itself is the main reason why Microsoft does not recommend using two or more controllers simultaneously. Nevertheless, scientists invented and published methods on combining signals from several Kinect devices [31,32,33]. It is noticeable that data gathered from the Kinect is incomplete, due to the lack of information about joint rotation along the bone. This is the result of the skeleton model design, where each of 20 tracked joints is described as a single point.

**Figure 3:**
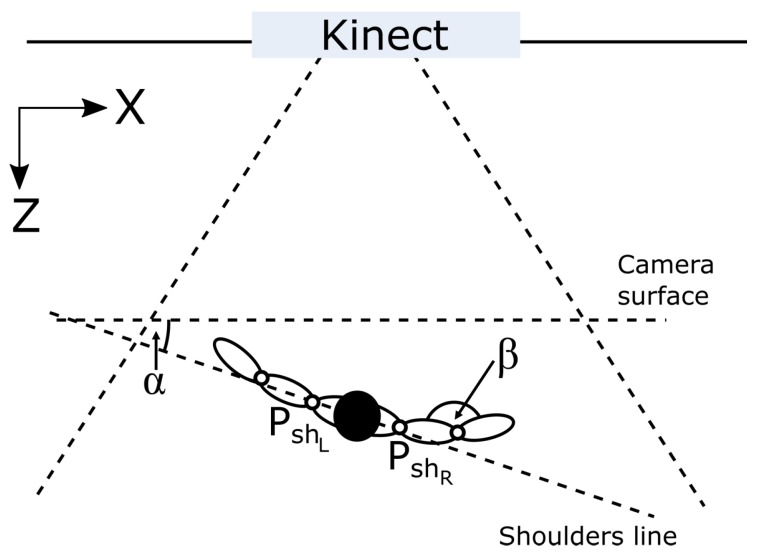
Rotation angle α between the user and Kinect.

The last major flaw of Microsoft Kinect controller is an occlusion, which occurs when a part of a user’s body is covered by another object or is hidden behind any other body part (self-occlusion). The occlusion by an external object seems obvious and does not require any additional explanation; however, self-occlusions are less intuitive. They are connected with Kinect’s sensitivity to user’s rotation to the camera (α angle in Figure 3), as Microsoft recommends working with Kinect in the face off pose, which is not precisely defined. Self-performed experiments allowed observing α angle changes while the user rotated in front of the camera. Assuming that $P_{Sh_{L}}=\left[p_{{Sh}_{L},X}^{K},p_{{Sh}_{L},Z}^{K}\right]$ is the position of the left shoulder and $P_{Sh_{R}}=\left[p_{{Sh}_{R},X}^{K},p_{{Sh}_{R},Z}^{K}\right]$ is the position of the right shoulder, both defined in a Kinect controller coordinating system (limited to *X* and *Z* axes in Figure 3), then α can be calculated according to Equation (3).

(3) $$\begin{array}{cc} \alpha & =\left\{\begin{array}{cc} atan(\frac{|p_{{Sh}_{R},Z}^{K}-p_{{Sh}_{L},Z}^{K}|}{|p_{{Sh}_{R},X}^{K}-p_{{Sh}_{L},X}^{K}|}) & ,|p_{{Sh}_{R},X}^{K}-p_{{Sh}_{L},X}^{K}|\neq 0,\\ \frac{\Pi }{2} & ,|p_{{Sh}_{R},X}^{K}-p_{{Sh}_{L},X}^{K}|=0. \end{array}\right. \end{array}$$

There are two possible strategies that Kinect uses when the occlusion happens. Either the device tries to estimate the location of the covered joint or, when it is not able to perform even rough estimation of the joint position, it stops tracking of such a joint. The results of self-performed experiments show that considerable occlusions occur when the user is rotated by more than $50{}^{\circ }$ ($\alpha >50{}^{\circ }$).

**Figure 4:**
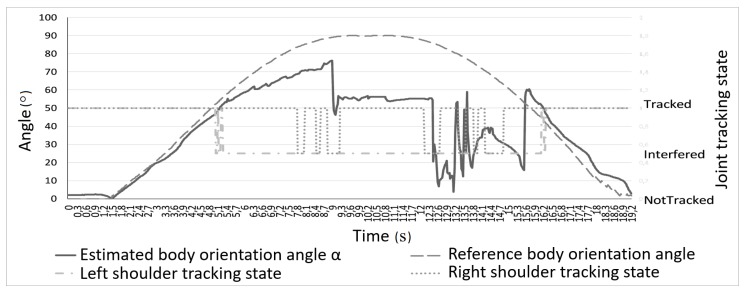
Shoulders joints tracking state in relation to body rotation angle α.

**Figure 5:**
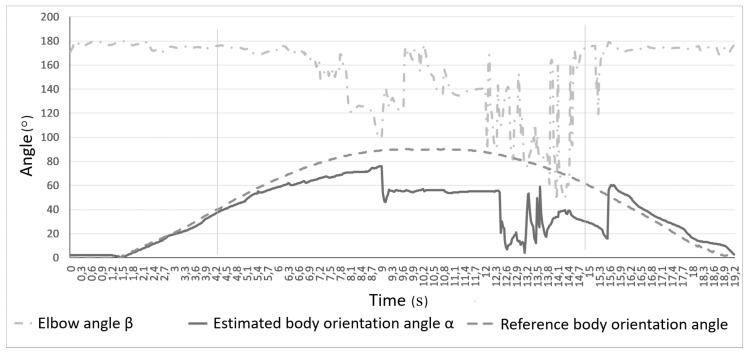
Elbow angle β estimation in relation to body rotation angle α.

Charts presented in Figure 4 and Figure 5 show measured joints tracking states and the elbow angle (angle β in Figure 3) changes during the rotation, respectively. In Figure 4, it is visible that tracking the states of both shoulders joints fluctuate between *Tracked* and *Interferred* states, when the subject was rotated more than $50{}^{\circ }$ ($\alpha >50{}^{\circ }$) in relation to Kinect’s camera. It is worth mentioning that even an easily visible right shoulder joint lost tracking state during that rotation. In Figure 5, it can be noticed that the estimation of the right elbow angle β was unstable for considerable body rotation ($\alpha >50{}^{\circ }$), though the hand was fully visible all the time.

### 2.2. IMU Characteristics

IMU devices have been in professional usage from decades. Within the scope of the current study, there are two types of sensors that measure inertial forces affecting them: accelerometers and gyroscopes. All experiments presented in this paper were performed with the usage of IvenSense MPU-6050 module (San Jose, CA, USA), which integrates both of these types of inertial devices and a thermometer. Even though the accelerometer and the gyroscope were integrated on a single PCB (Printed Circuit Board), their data must be processed individually, as they are affected by the different set of external noises with various frequencies that must be filtered out to make them usable. The IMU device used in our research can be classified according to [34] as an industrial device. Characteristics described in the article are thus representative for other devices belonging to this class.

**Figure 6:**
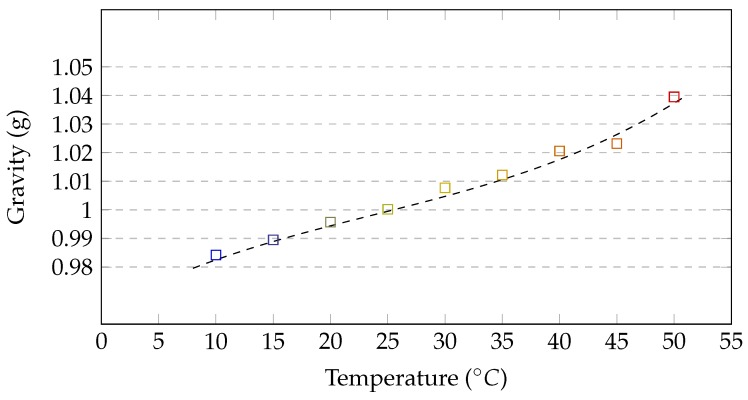
Accelerometer gravity measurements in temperature range 10–50 ${}^{{}^{\circ}}$C.

An accelerometer is an inertial sensor that measures linear forces, affecting it along the device coordinating system axes. Usually, the measurements are defined in relation to the gravity force (1 g ≈ 9.81 m/s${}^{2}$), so it is possible to estimate its linear acceleration during the motion. When the device rests, the theoretically correct measurement should be 1 g in the upward direction and 0 g in two other directions. However, due to external noises (mainly high frequency), the resting device measurements are oscillating around theoretical values. The analysis of these oscillations, during calibration, allows for designing low pass filter [35,36] that would be able to filter out at least major part of these high frequency noises. Accelerometers are also sensitive to operating temperature changes and its influence on sensor’s accuracy was the subject of some researches [37,38]. The temperature sensitivity is caused by the architecture of the sensor, which is built with several capacitors, hence the operating temperature has influence on their capacity. During the self conducted experiment, the measurement of the g-force was observed when the resting device was heated and then cooled down multiple times in the temperature range 10–50 ${}^{{}^{\circ}}$C. The results of this experiment are presented in Figure 6. This chart shows that the measurement tends to change from under to overestimation with the temperature change. It is also worth noticing that the measured value of the gravity is up to 4% different than the expected, what may influence the further results. According to the MPU-6050 specification and results of self-conducted experiment, the neutral operating temperature for the considered module is 25 ${}^{{}^{\circ}}$C. Due to the fact that the operating temperature (*T*) of the IMU device, placed on a human body, rises and stabilizes at approx. 30 ${}^{{}^{\circ}}$C, some sort of compensation (*f(T)*) is required there. Equation (4) presents the formula of the measured error:

(4) $$f\left(T\right)=7\cdot 10^{-7}T^{3}-5\cdot 10^{-5}T^{2}+0.0022T+0.9648.$$

A gyroscope—the second type of inertial sensors—measures its angular velocity in deg/s units. If the sensor is in the resting state, all measurements should equal 0 for each of three axes. However, the device suffers from the long-term bias that should be limited (ideally–removed). The analyzed noise has low frequency characteristics, thus the appropriate high pass filter [39], limiting its influence, should be applied. As a MEMS (MEMS—microelectromechanical system) based gyroscope is also built from multiple capacitors, their bias is influenced by the temperature as well. However, as the distortions related to the temperature have low frequency nature, their influence on the data is reduced during signal filtration by a high-pass filter.

**Figure 7:**
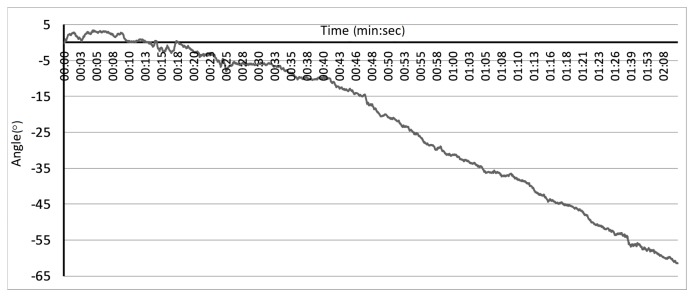
Gyroscope drift for non-moving devices.

The last problem, considered by the authors, related to IMU devices is the incompleteness of the data provided by these units. To calculate the accurate orientation of the IMU module, data from the accelerometer and the gyroscope (orientation around corresponding axes) should be fused together. The accelerometer allows for calculating orientation around two axes, even though it measures forces along all three orthogonal directions. The orientation (or the rotation) around the gravity vector is unmeasurable for such device. Theoretically, the gyroscope should retrieve orientation measures around all three directions; however, even the filtered signal contains some errors. Its numerical integration over time results in the significant data drift that makes such measurements useless. An example of the data drift is presented in Figure 7. Despite the sensor remaining motionless during the experiment, the estimations based on the numerically integrated data showed the sensor theoretical rotation about $60{}^{{}^{\circ}}$ within 2 min (about 0.5${}^{{}^{\circ}}$/s).

### 2.3. Hybrid, Orientation Based, Human Limbs Motion Tracking Method

**Figure 8:**
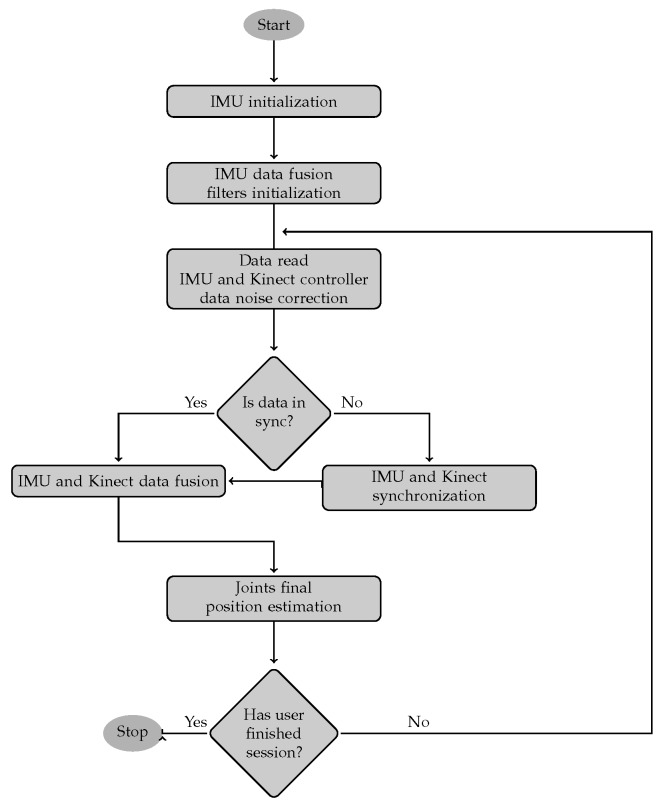
Orientation based data fusion method schema.

As an input to the method, two (Kinect and IMU), independently registered, streams of data representing tracked human skeleton bone oations, were considered. The problem that the motion tracking method needs to face was estimating human skeleton selected joint (*j*) fused (*F*) from considered data sources, position ($P_{j,t}^{F}={\left[p_{j,x}^{F},p_{j,y}^{F},p_{j,z}^{F}\right]}_{t}$) within the referenced three-dimensional coordinating system, in a particular moment of time (t). To solve this problem, the authors propose a novel, hybrid human limbs motion tracking method, based on the continuous linear fusion of skeleton bone oations (contrary to state-of-the-art skeleton joint positions fusion) with respect to the current motion context. It takes into consideration the controllers’ reliability and compensates measurement devices’ imperfections described in previous chapters. The data fusion method has been split into several, sequential phases presented on a diagram in Figure 8 and described in the further part of this article.

The discussed novel data fusion method can be presented as a general formula with Equation (5):

(5) $$P_{j,t}^{F}=f\left(A,G,T,P_{j-1,t}^{K},P_{j,t}^{K},\Delta t,P_{j-1,t}^{F},P_{sh_{L},t}^{K},P_{sh_{R},t}^{K},l\right).$$

**Figure 9:**
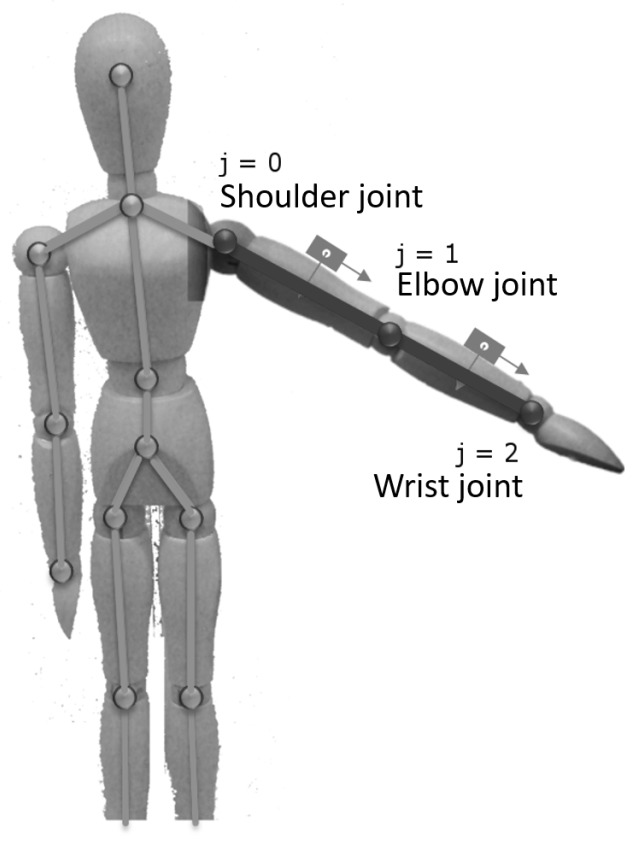
The hand joints simplified hierarchical model.

The estimation of the selected joint *j* position ($P_{j,t}^{F}$) in particular time *t* is based on the measurements of the accelerometer ($A=[a_{x},a_{y},a_{z}]$), the gyroscope ($G=[g_{x},g_{y},g_{z}]$) and the positions of the selected joint and its parent, measured by the Kinect controller ($P_{j,t}^{K}={\left[p_{j,x}^{K},p_{j,y}^{K},p_{j,z}^{K}\right]}_{t}$, $P_{j-1,t}^{K}={\left[p_{j,x}^{K},p_{j,y}^{K},p_{j,z}^{K}\right]}_{t}$). Additionally, to compensate for imperfections of IMU measurements, the current operating temperature *T* is taken into consideration. In addition, positions of both limb (i.e., shoulder) joints ($P_{sh_{L},t}^{K},P_{sh_{R},t}^{K}$) are used to determine the reliability of the data and choose the right data fusion formula. The discussed method estimates the joint position with some time interval Δt aligned to the Microsoft Kinect update interval. IMU are stuck to the body surface, on the bone of length *l*, between the tracked joint *j* and its parent joint j−1. To define the fused position $P_{j,t}^{F}$ in superior coordination space, it has to be aligned to the fused position of its parent joint $P_{j-1,t}^{F}$. The simplified hierarchical model of the limb (described on a hand example), as well as the placement of IMUs, are presented in Figure 9.

#### 2.3.1. Coordination Space

**Figure 10:**
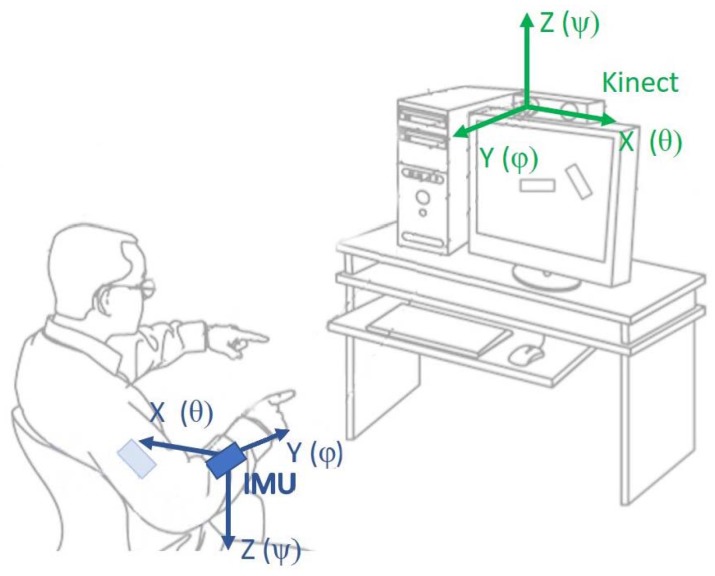
Measurement devices’ coordination spaces.

Both measurement devices have different local coordination spaces that presented in Figure 10.

As the majority of the data processed in the method are gathered from the Kinect controller, the global coordination space equals the Kinect’s coordination system. This allows for reducing the number of required transformations and to improve the overall performance of the system. To unify both coordination spaces, frame of reference needs to be defined according to which any further rotations are estimated. During the calibration process, when a user stands without motion in T-pose in front of a Kinect camera, such frame of reference is defined. Later, during motion, each IMU estimation (${\left[x,y,z\right]}_{I}^{T}$) needs to be transformed into the Kinect’s coordination space (${\left[x,y,z\right]}_{K}^{T}$). As an IMU coordination system is anchored to the gravity vector, and retains its orientation regardless of device rotations, such transformation is defined as a sequence of a rotation by $180{}^{{}^{\circ}}$ around the *y*-axis ($R_{Y}\left(180\right)$) and scale (*S*) transformations according to Equation (6). A definition of one common coordination space for both measurement devices is necessary to fuse their signals:

(6) $${\left[\begin{array}{c} x\\ y\\ z \end{array}\right]}_{K}=R_{Y}\left(180\right)S{\left[\begin{array}{c} x\\ y\\ z \end{array}\right]}_{I}=\left[\begin{array}{ccc} \cos(180) & 0 & \sin(180)\\ 0 & 1 & 0\\ -\sin(180) & 0 & \cos(180) \end{array}\right]\left[\begin{array}{ccc} 1 & 0 & 0\\ 0 & -1 & 0\\ 0 & 0 & 1 \end{array}\right]{\left[\begin{array}{c} x\\ y\\ z \end{array}\right]}_{I}=\left[\begin{array}{ccc} -1 & 0 & 0\\ 0 & -1 & 0\\ 0 & 0 & -1 \end{array}\right]{\left[\begin{array}{c} x\\ y\\ z \end{array}\right]}_{I}.$$

#### 2.3.2. IMU Initialization

**Figure 11:**
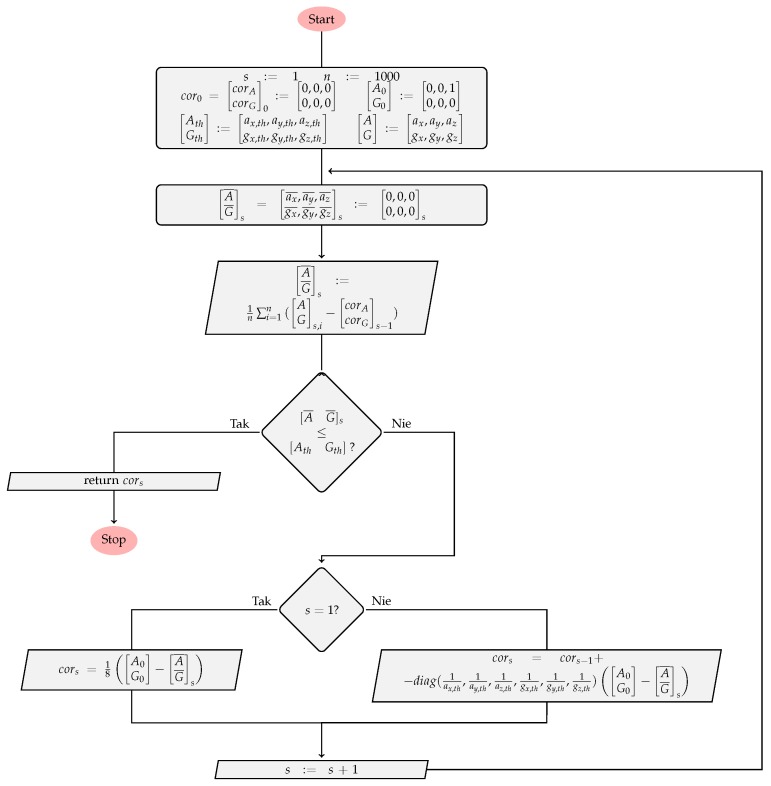
Diagram of the inertial measurement unit (IMU) initialization routine.

The goal of the IMU initialization procedure, presented in Figure 11, is to calculate the data (G,A) correction factors matrix ($cor={\left[cor_{A}\;cor_{G}\right]}^{T}={\left[\left[c_{a}x,c_{a}y,c_{a}z\right]\;\left[c_{g}x,c_{g}y,c_{g}z\right]\right]}^{T}$) for each IMU individually.

During this initialization, IMU modules must lay down without any motion, in the face-up position, as this is the position, in which we know exactly the expected data ($A_{0}=\left[0,0,1\right]$ for the accelerometer and $G_{0}=\left[0,0,0\right]$ for the gyroscope). However, the ideal values are impossible to achieve due to the noise that cannot be completely removed. Because of this, the calibration routine works iteratively (*s*—iteration index) as long as the average IMU measurement errors (${\left[\bar{A}\;\bar{G}\right]}^{T}={\left[\left[\bar{a_{x}},\bar{a_{y}},\bar{a_{z}}\right]\;\left[\bar{g_{x}},\bar{g_{y}},\bar{g_{z}}\right]\right]}^{T}$) are lower than the defined threshold (${\left[A_{th}\;G_{th}\right]}^{T}={\left[\left[a_{x,th},a_{y,th},a_{z,th}\right]\;\left[g_{x,th},g_{y,th},g_{z,th}\right]\right]}^{T}$). In this case, a relation of ${\left[\bar{A}\;\bar{G}\right]}^{T}\leq {\left[A_{th}\;G_{th}\right]}^{T}$ is true when each element of the first matrix is lower than the corresponding element of the second matrix. As a result of this calibration method, the matrix cor is calculated, which elements added to the current IMU data, allow to use data with the desired accuracy.

If ${\left[A\;G\right]}_{s,i}$ is an *i*–th out of the *n* consecutive IMU device measurements for the iteration *s*, then the average error ${\left[\bar{A}\;\bar{G}\right]}^{T}$ is calculated according to Equation (7):

(7) $${\left[\begin{array}{c} {\bar{A}}^{T}\\ {\bar{G}}^{T} \end{array}\right]}_{s}=\left\{\begin{array}{cc} \frac{1}{n}\sum_{i=1}^{n}{\left[\begin{array}{c} A^{T}\\ G^{T} \end{array}\right]}_{s,i}, & s=1,\\ \frac{1}{n}\sum_{i=1}^{n}{\left[\begin{array}{c} A^{T}\\ G^{T} \end{array}\right]}_{s,i}-{\left[\begin{array}{c} cor_{A}^{T}\\ cor_{G}^{T} \end{array}\right]}_{s-1}, & s>1. \end{array}\right.$$

Then, the data correction factors matrix cor, for single measurement, is defined by Equation (8):

(8) $$cor_{s}={\left[\begin{array}{c} cor_{A}^{T}\\ cor_{G}^{T} \end{array}\right]}_{s}=\left\{\begin{array}{cc} \frac{1}{8}\left(\left[\begin{array}{c} A_{0}^{T}\\ G_{0}^{T} \end{array}\right]-{\left[\begin{array}{c} {\bar{A}}^{T}\\ {\bar{G}}^{T} \end{array}\right]}_{1}\right), & s=1,\\ cor_{s-1}-diag\left(1/a_{x,th},1/a_{y,th},1/a_{z,th},1/g_{x,th},1/g_{y,th},1/g_{z,th}\right)\left(\left[\begin{array}{c} A_{0}^{T}\\ G_{0}^{T} \end{array}\right]-{\left[\begin{array}{c} {\bar{A}}^{T}\\ {\bar{G}}^{T} \end{array}\right]}_{s}\right), & s>1. \end{array}\right.$$

#### 2.3.3. IMU Data Fusion Filters Initialization

The Madgwick filter [13], defined with the formula presented in Equation (9), has been used as an IMU module inherent accelerometer and gyroscope data fusion method:

(9) $$Q^{I}=m\left(A,G,f_{m},\Delta t\right).$$

This filter estimates the IMU module orientation in the quaternion form ($Q^{I}$), using accelerometer data (*A*), gyroscope data (*G*) and the filtration factor ($f_{m}$) based on the gyroscope drift characteristics. Madgwick filter processes the IMU data with the time interval Δt. Before the filter can run on the real data, it needs to be initialized to set up its internal parameters correctly. Madgwick filter initialization requires running the filter multiple times for the measurements of non-moving accelerometer and gyroscope sensors. During this initialization the filtration factor $f_{m}$ needs to be set to the relatively high value e.g., $f_{m}=2$. After the completion of the initialization process, $f_{m}$ should be defined according to the average Angle Random Walk (ARW) noise value $\tilde{\omega }$ that can be calculated with the Allan’s variance [40,41,42]. In case of exploited MPU-6050, IMU modules filtration factor $f_{m}$ is calculated according to the original article [13] equation: $f_{m}=\sqrt{\frac{3}{4}\tilde{\omega }}$, and equals 0.082.

#### 2.3.4. IMU and Kinect Controller Data Noise Correction

The data noise correction is a crucial phase for the data fusion. Its goal is to remove as much noise as possible from the raw data. All methods used in this phase are related to measurement devices characteristics described earlier in this article.

The first step to improve the IMU module data quality is the accelerometer measurements (*A*) error compensation due to the device operating temperature (*T*). For the neutral temperature ($T_{0}$ = 25 ${}^{{}^{\circ}}$C) and the correction factor $f_{T}=0.0011$, the correction is defined with Equation (10). The value of $f_{T}$ has been estimated in self-conducted experiments:

(10) $$A^{\prime }=A/\left(1+f_{T}\left(T-T_{0}\right)\right).$$

The low and high pass filtration of accelerometer and gyroscope data is a part of Madgwick filter implementation, so there is no need to repeat this process. The second measurement device—Kinect controller—requires two data correction steps in order to improve the measurement quality: the correction of the distance estimation and smoothing the joint position estimations. The distance estimation correction is done by the function presented in Equation (11), which is the opposite of the distance estimation error model (Equation (1)). The argument of this function (*z*) is directly taken from the Kinect’s distance estimation API:

(11) $$f\left(z\right)=z^{\prime }=-0.02z^{3}+0.11z^{2}-0.27z+0.25.$$

The smoothing of the Kinect’s joint positions ($P_{j,t}^{K}$) is necessary as coordinates’ fluctuations may happen, especially when occlusions occur. While there is an occlusion, the position of the same joint in two consecutive frames (measurements) can differ few centimeters, which is physically and anatomically impossible, while refresh rate of the Kinect is 30 Hz. The smoothing can be done by the Kinect’s firmware, but this approach gives a significant delay of the signal interpretation. The alternative approach to positions smoothing, which was exploited in the method, is a simple low pass filter. The method uses the 1st order exponential filter to remove unreliable positions estimations defined with Equation (12) and the filtration factor $f_{LPF}=0.065$:

(12) $${P^{\prime }}_{j,t}^{K}=f_{LPF}\cdot P_{j,t}^{K}+\left(1-f_{LPF}\right)\cdot {P^{\prime }}_{j,t-1}^{K}.$$

**Figure 12:**
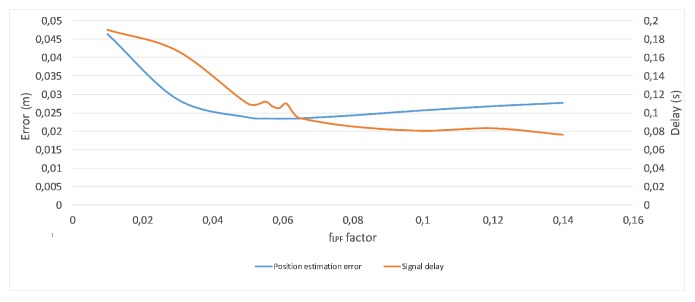
Estimation error and signal delay of smoothing function charts.

The value of $f_{LPF}$ has been chosen as a compromise between error of estimation and smoothed signal delay. Figure 12 presents charts of estimation error and signal delay related to $f_{LPF}$ factor of proposed smoothing function.

#### 2.3.5. IMU and Kinect Synchronization

The data fusion of any two signals requires signal alignment in the time domain. This guarantees fusion of samples that were collected in the same time synchronously. The goal of IMU and Kinect signals synchronization is to find the time offset τ between two data streams. To synchronize both signals, they must have the same frequency, so the IMU signal has to be downsampled from 100 Hz to 30 Hz, which is a nominal frequency of the Kinect controller. The downsampling has been implemented in the form of decimation, where Kinect’s new sample availability defines the time of IMU sample picking. Then, the time offset τ between the IMU signal *I* and the Kinect signal *K* was defined as a time offset argument of the cross-correlation algorithm that gives the maximum value of correlation between these two signals (variable $\tau_{max}$) (Equations (13a) and (13b)). The value of $\tau_{max}$ is added to the timestamp of IMU samples to align it to the Kinect’s signal:

(13a) $$\begin{array}{cc} \left(I*K)(\tau \right) & =\int_{-\infty }^{+\infty }I\left(t\right)K\left(t+\tau \right)dt, \end{array}$$

(13b) $$\begin{array}{cc} \tau_{max} & =\underset{\tau }{argmax}\left(\left(I*K\right)\left(\tau \right)\right). \end{array}$$

#### 2.3.6. IMU and Kinect Data Fusion

The data fusion method processes time aligned filtered data and takes into consideration their reliability. The most serious concerns about the quality of data used within the fusion were related to the data provided by Microsoft Kinect controller. The decision about Kinect’s data quality was based on the several information gathered during the motion. The first one is the combination of the joint tracking state and the noise level of its position estimation. A fully visible joint has its state set to *Tracked* value and its position can be considered as reliable. Otherwise, if the tracking state value is set to *Interferred*, the position estimation noise level must be calculated to check if the value can be treated as reliable or not. In the *Interferred* state, the position of the joint is estimated basing more on the predication than direct measurements, so its accuracy might be low and values may differ significantly between consecutive frames (or in a short time period). Other parameters taken into consideration are: the value of the angle α between the user and the camera, as well as the information about the tracking state of both shoulder joints. Microsoft Kinect has been designed to track the motion of the human who stands frontally to the camera. If the user rotates too much, Kinect is not able to track the motion correctly anymore. During self experiments, it turned out that the maximum reliable angle between the human and the Kinect is $50{}^{{}^{\circ}}$. Exceeding this rotation angle results in unreliable, often random, values of all joint positions. As the value of the α angle is calculated using position values of both shoulder joints, both of them should be fully visible (tracking state set to *Tracked*), and their position values estimations should be stable in time. This means that the angle measurement standard deviation should be lower than $1.5{}^{{}^{\circ}}$, so there should be no rapid changes in shoulders position estimations, which are characteristic for the significant error in the provided data.

If all of the mentioned conditions are satisfied, signals are fused according to Equation (14). The novelty of the presented method, in comparison with literature approaches, lies in the fact that the conditionally fused data represents skeleton bone oations in the form of Euler angles $E=\left[\begin{array}{ccc} \phi & \theta & \psi \end{array}\right]$, instead of joints resulting positions. Microsoft Kinect controller provides natively information about selected bone oation in the form of quaternion, calculated using current bone’s joint positions. Used in the presented method, Madgwick’s filter also provides IMU orientation as single quaternion due to the fact that orientation fusion, described in this article, was defined with Euler angles. Quaternions from both sources need to be converted to this form before they are fused. To prevent the Euler angle’s singularity problem, during the conversion from quaternion, an additional check is performed. This step is supposed to detect if the sensor is oriented vertically up or down and, if it is, convert data to fixed values. The singularity is detected by the sum of multiplications of quaternion’s values:

$$c_{s}=q_{X}q_{Y}+q_{Z}q_{W}.$$

If the value of $c_{s}$ is greater that 0.4999, it means that the sensor is oriented vertically up, and, if it is lower than −0.4999, then it is oriented down. In case the singularity appears, the ϕ,θ,ψ are defined as:

- ϕ=0,
- $\theta =\pm 2\tan^{-1}\left(q_{X}/q_{W}\right),$
- ψ=±Π/2.

The ± is related to the sign of the $c_{s}$ value. The proposed data convertion method is based on the assumption that we need to align estimations to some fixed value around the singularity point. This means that we lose real measurements. On the other hand, ±0.4999 threshold value cuts off the orientation angle with a value exceeding $\pm 87{}^{{}^{\circ}}$. Details about the conversion from the quaternion to the Euler angles can be found in [43]:

(14) $$E_{t}^{F}={\left[\begin{array}{c} \phi^{F}\\ \theta^{F}\\ \psi^{F} \end{array}\right]}_{t}=diag\left(w_{\phi },w_{\theta },w_{\psi }\right){\left[\begin{array}{c} \phi^{I}\\ \theta^{I}\\ \psi^{I} \end{array}\right]}_{t}+diag\left(1-w_{\phi },1-w_{\theta },1-w_{\psi }\right){\left[\begin{array}{c} \phi^{K}\\ \theta^{K}\\ \psi^{K} \end{array}\right]}_{t}.$$

**Figure 13:**
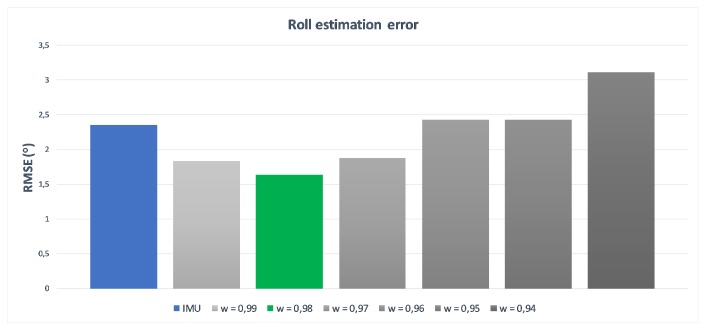
Influence of $w_{\phi }$ to the roll rotation estimation error–roll for Kinect is not available.

**Figure 14:**
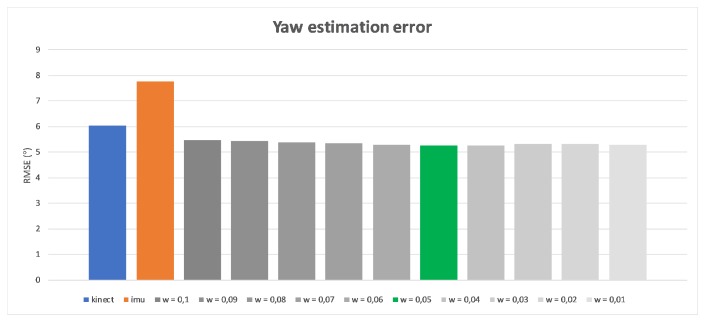
Influence of $w_{\theta }$ to the yaw rotation estimation error.

**Figure 15:**
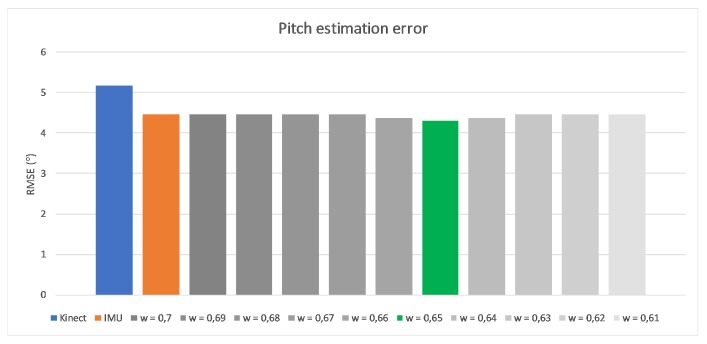
Influence of $w_{\psi }$ to the pitch rotation estimation error.

The originally elaborated weights $\left[w_{\phi },w_{\theta },w_{\psi }\right]$ describe the importance level of IMU measurements. They were based both on the device precision and the data completeness, and were defined as [0.98,0.05,0.65], respectively. The very high and very low first and second values are the result of measurement devices’ characteristics described in the beginning of this article. As Kinect is not able to detect Roll rotation, the estimation of IMU is promoted (the first value). Similarly, the second value that relates to the Yaw rotation, for which IMU estimation is considerably less accurate than Kinect’s estimation, has been depreciated with a low second coefficient value. The Pitch rotation can be estimated by both devices; this is why the third value is defined close to 0.5. Exact values are the results of self-made research about estimating arm and forearm bone oations by both measurement devices while the hand motion was performed only in one, selected degree of freedom. Such constraint guaranteed that possible, accidental rotations around other axes than the considered one have no impact on gathered results. Figure 13, Figure 14 and Figure 15 present charts of the influence of *w* factor’s value to the estimation error of each rotation during mentioned experiments.

If Kinect’s data turn out to be unreliable, they cannot be fused with the IMU data. Averaged bone oations are then estimated basing on the previously (t−1) fused estimation and the current change of IMU orientation, according to Equation (15):

(15) $$E_{t}^{F}={\left[\begin{array}{c} \phi^{F}\\ \theta^{F}\\ \psi^{F} \end{array}\right]}_{t}={\left[\begin{array}{c} \phi^{F}\\ \theta^{F}\\ \psi^{F} \end{array}\right]}_{t-1}+diag\left(w_{\phi },w_{\theta },w_{\psi }\right)\;\left({\left[\begin{array}{c} \phi^{I}\\ \theta^{I}\\ \psi^{I} \end{array}\right]}_{t}-{\left[\begin{array}{c} \phi^{I}\\ \theta^{I}\\ \psi^{I} \end{array}\right]}_{t-1}\right).$$

However, in the case of Kinect data unreliability, weights $\left[w_{\phi },w_{\theta },w_{\psi }\right]$ are defined differently. In Equation (15), these weights were defined as

$$[0.98,\left(1-t_{noise}/10\right)\cdot 0.65,\left(1-t_{noise}/10\right)\cdot 0.65],$$

where $t_{noise}$ coefficient represents the time in seconds, when Kinect stayed unreliable and its maximum accepted value is set to 10s. After this time, the estimation of the new bone orientation (only around two axes) stops, until Kinect is available again, and the method privileges the IMU signal. In this scenario, as the IMU is in fact the only source of up to date data, the initial coeficient of Yaw rotation ($w_{\theta }$) has been increased compared to the value in the scenario when both Kinect and IMU data are available to fuse. Leaving this value on the low level caused the rotation to not be detectable.

#### 2.3.7. Joints Final Position Estimation

The final joint position ($P_{j,t}^{F}$) estimation requires the information regarding the position of the joint’s parent in the hierarchical skeleton model ($P_{j-1,t}^{F}$), the estimated while calibration (and fixed) length of the bone between the current joint and its parent (*l*) and the bone orientation estimation ($E_{t}^{F}={\left[\phi^{F},\theta^{F},\psi^{F}\right]}_{t}$). As a result of the data fusion, estimated orientations were presented in the form of Euler angles. However, this representation of the orientation may complicate further calculations. To simplify them, the opposite conversion from Euler angles to the quaternion $Q_{t}^{F}$ is recommended, according to the formula described in [43]. Since the skeletal model is defined as a hierarchical structure, the newly estimated joint position must be aligned to the position of its parent joint. Taking that into consideration, the final joint position calculation procedure is described with Equation (16):

(16) $$P_{j,t}^{F}=P_{j-1,t}^{F}+Q_{t}^{F}\cdot \left[l,0,0\right]\cdot {Q_{t}^{F}}^{-1}.$$

## 3. Results

In order to verify the accuracy of the elaborated method, several experiments were performed. They were conducted with the Vicon motion capture system, as a source of ground truth reference data. Hand movements of three subjects were monitored, being tracked simultaneously with Kinect controller, two IMUs attached to the hand’s arm and forearm bones on the skin surface, as well as the Vicon tracking system. In current implementation of the proposed method, we have made the assumption that each sensor is statically assigned to the particular limb. This means that dedicated (to arm and forearm) sensors are assigned to arm and forearm, respectively, and can not be misplaced. Automatic sensors identification and their relative hierarchical assignment leave room for further method improvements.

**Figure 16:**
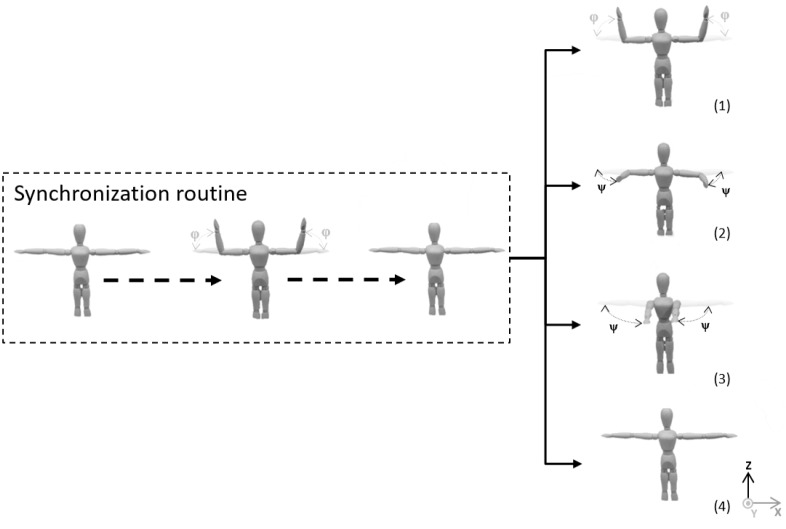
Movement sequences performed during tests.

Each user had to perform four exercises, each of them twice with five repetitions per single try. Each try started with the devices’ synchronization in the T-Pose, and then five repetitions of the motion began. The initial T-pose has been used as a frame of reference and joint positions were updated according to this position. Exercises focused on the horizontal and vertical hand flexion in the elbow joint, horizontal hand motion and keeping a straight arm in the T-pose with no motion for at least 60 s. All of these exercise schemes are presented in Figure 16.

Three out of four motion trajectories used in the verification experiments can be considered as difficult according to the measurement devices’ characteristics.The first motion is relatively easy to track by both devices as no joints occlusion was observed there, and it was fully measurable by the accelerometer as well as the gyroscope. The second and the third trajectories define motions around the axis, where the IMU device was not able to correctly measure current orientation and Kinect controller loses visibility of the joint due to the self occlusion. The important difference between these two motions was the number of occluded joints: the elbow only in the second motion and the elbow and the shoulder in the third. This difference had an impact on Kinect’s estimations reliability, which was worse when the shoulder was not fully visible to the controller’s camera. The last motion focused on IMU’s constant measurement drift around the axis perpendicular to the gravity vector. Mentioned physical body movements let us claim that selected exercises represent the most challenging trajectories that the motion tracking system can face, so it should be able to track other trajectories with similar or better accuracy. The pace of the movements was characteristic for the movements of rehabilitation’s physical exercises and the subjects could perform the motions according to individual preferences, without any specific constraints. The only formal pace limitation is the Microsoft Kinect tracking performance (Kinect has 30 Hz slower operating frequency than the exploited IMU controller).

For the self made measurement device used in my studies, both IMU work with different addresses in I2C communication channel and, in order to simplify the code and processing in the current version of the method, sensors are statically assigned to the limbs/bones. It means that the sensor with the channel address *addr1* should always be attached to the upper arm and *addr2* to the forearm (or generally *addr1* to the parent bone and *addr2* to its child).

The accuracy of three parameter estimations has been observed: the position of the elbow joint, the position of the wrist joint and the angle between the arm and the forearm measured in the elbow joint. Those values have been compared with the corresponding parameters estimated with Kalkbrenner’s method [14], which, on the contrary, fused positions instead of orientations of selected joints. The position estimation error has been defined as the root mean squared error (RMSE) of the Euclidean distance ($d_{e}$) between joint reference position, measured by the Vicon motion capture system ($P_{j}^{V}$), and joint position estimated by two competitive data fusion methods. The first one is the joint position-based Kalkbrenner data fusion method ($P_{j}^{FP}$) and the second: the bone rotation-based fusion method ($P_{j}^{FO}$) proposed by the authors of this article. The RMSE value has been calculated with Equation (17) [44]. The error for the elbow joint angle β estimation has been calculated in a similar way:

(17) $${RMSE}_{j}^{F}=\sqrt{\frac{1}{n}\sum_{i=1}^{n}d_{e}{\left(P_{j,i}^{V},P_{j,i}^{Fs}\right)}^{2}}\;,s=\left\{O,P\right\}.$$

**Figure 17:**
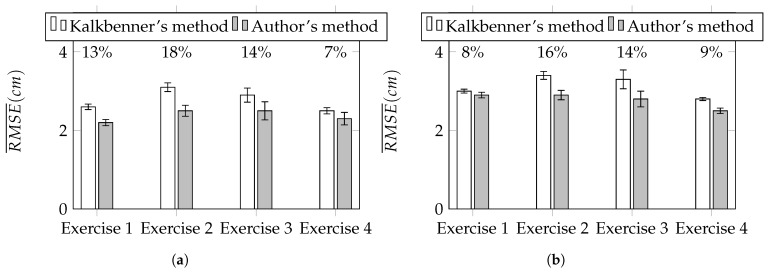
Average root square mean error $\bar{RMSE}$ of the elbow (**a**) and the wrist (**b**) joint position estimation.

**Figure 18:**
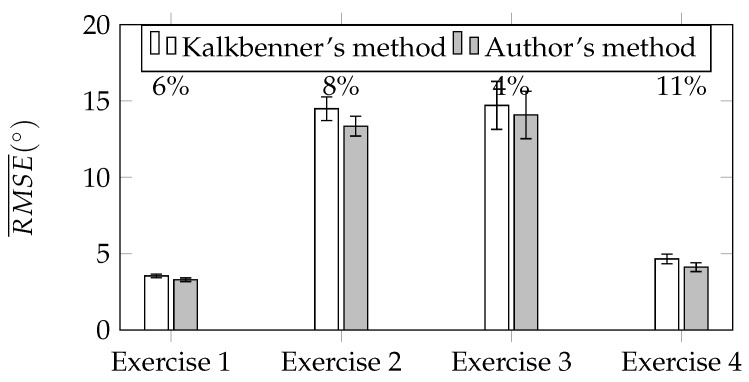
Average root square mean error $\bar{RMSE}$ of the elbow flexion angle β.

The overall error of both methods, for each test exercise, has been calculated as a mean of RMSE defined with eqation

$$\bar{RMSE}=\frac{\sum_{i=1}^{n}RMSE}{n}$$

of each motion tracking session. To compare Kalkbrenner’s method with the authors’ novel method, the ratio *r* between the difference of $\bar{RMSE}$ of both methods to the $\bar{RMSE}$ of Kalkbrenner’s method has been calculated according to Equation (18) and expressed in the form of percents. Figure 17a,b show the summary of $\bar{RMSE}$ for tracked upper limb joints: the elbow and the wrist. Above each pair of charts’ bars, the value of *r* ratio has been presented. Based on the results presented in Figure 17a,b, the improvement of the proposed orientation-based fusion method, in reference to the best in the literature reference—Kalkbrenner’s method, has been noticed. However, the improvement ratio varies between joints. The error of the elbow joint position estimation has been reduced up to 18% (from about 3.1 cm to 2.6 cm) and the wrist joint position estimation error up to 16% (from about 3.4 cm to 2.9 cm). The difference in the improvement ratio between these two joints is caused by the fact that the elbow joint is closer to the skeleton model root than the wrist joint, so it accumulates less ascending joint position estimation errors. Figure 18 presents the chart of $\bar{RMSE}$ of the elbow angle β estimation (calculated similarly to Equation (17)). In this value estimation, the improvement has been also noticed and its ratio is up to 11% (from about $4.6{}^{{}^{\circ}}$ to $4.1{}^{{}^{\circ}}$):

(18) $$r=\frac{\bar{RMSE_{j}^{P}}-\bar{RMSE_{j}^{O}}}{\bar{RMSE_{j}^{P}}}.$$

## 4. Discussion and Conclusions

The authors presented a new, orientation-based method for skeleton joint positioning, which compensates devices’ imperfections and considers the context of the motion. It was tested on a set of right hand movements, which appeared to be demanding for measurement devices. Results were compared with those gathered from the position-based fusion method and the reference, professional, ground truth Vicon tracking system. Presented results prove that the newly elaborated, complex approach reduces joint position estimation error. The principle reason of such estimation improvement, identified by the authors, is related to the better stability of bone rotation estimation in reference to the position-based method, as well as the diminished inaccuracy of bone length measurements and the lack of the fixed skeleton model definition support. Moreover, the authors have found that the introduced complementary fusion of the signals, measured by both devices, reduces the influence of source signals’ imperfections on the final result. In the case of IMU devices, the information about their 3D space orientation (for IMU devices stuck to the body, this orientation also represents the orientation of the bone) was the default and required less complex data processing. The estimation of the joints’ positions, based on IMU measurements, required additional measurement of the selected bone length and additional geometric transformations (geometric transformations required to estimate the joint position based on IMU measurement has been described in detail in [14]), which have an impact on the final joint position estimation error. Considering the Microsoft Kinect controller, the human body skeletal model includes information about each bone length and its orientation. However, these length values are updated continuously and may differ significantly between two consecutive measurement frames. The difference might be even a few centimeters. During experiments, the measured differences in bone length were up to 5 cm for *Tracked* state joints. Moreover, even if the occlusion doesn’t occur, the estimation of angles between selected bones turns to be more stable in time than the estimation of joint positions. This means that the estimation of bone rotation is more reliable than joint position estimation. Furthermore, the bone rotation is the information available from both signal sources with minimal raw data processing, which has an impact on the quality of data used in the fusion.

What differentiates the proposed method from the already known methods described in the literature is the fact that it takes into consideration characteristics of the classes of chosen measurement devices, and provides the complex compensation of detectable errors that exist in their data. It also allows for improving the quality of the source data before their fusion. It is important that many of the characteristics described in this article adaptively respect the context of the performed motion. As an example, the variable accuracy of the distance measured between Kinect and the user, or the human rotation angle relative to the surface of Kinect, has an impact on the stability and reliability of Kinect’s data.

The goal of the conducted research was to find the method of human limbs motion tracking, which would take into consideration elaborated characteristics and would reduce joint position estimation error significantly. The proposed method, tested on the example of the upper limbs motion, has met this goal. The results obtained by the authors’ method have been compared with the results of the position-based data fusion method with the highest declared accuracy. Three parameters estimated by both methods have been compared: the elbow joint position error, the wrist joint position error and the elbow angle β error. The set of motions (Figure 16) used to test these methods have been chosen to check how methods work when source data is of poor quality due to the limitations and imperfections of measurement devices.

The results of the comparison of the elbow joint position estimation error are presented in the chart in Figure 17a. It shows that the achieved improvement in this parameter estimation is up to 18%. The comparison of the wrist joint position estimation error is presented on the chart in Figure 17b and the improvement of this parameter estimation is slightly lower than the improvement of the elbow joint position estimation error and its value is up to 16%. The difference in these values is caused by the distance between each of these joints and the root of the skeletal model. The joint that is further from the root accumulates existing errors of all joints that are defined between the root and itself. This explains why the improvement of the estimation error is lower for the wrist than for the elbow. The chart presented in Figure 18 shows the results of the comparison of the third parameter, which is the elbow angle estimation error during the motion. The best improvement in this parameter estimation was close to 11%. However, in exercises 2 and 3, which might be considered as the most difficult from the data quality detection point of view, the improvement is noticeably lower.

Obtained results prove that the novel data fusion approach, based on skeletal bone rotations, might be considered as an improved alternative to the well-known, joint position-based methods.

## Abbreviations

The following abbreviations are used in this manuscript:

RMSE	Root Mean Squared Error
IMU	Inertial Measurement Units
LPF	Low Pass Filter
